# Quantifying and Tracing Information Cascades in Swarms

**DOI:** 10.1371/journal.pone.0040084

**Published:** 2012-07-12

**Authors:** X. Rosalind Wang, Jennifer M. Miller, Joseph T. Lizier, Mikhail Prokopenko, Louis F. Rossi

**Affiliations:** 1 CSIRO Information and Communication Technologies Centre, Marsfield, New South Wales, Australia; 2 Department of Mathematical Sciences, University of Delaware, Newark, Delaware, United States of America; 3 Max Planck Institute for Mathematics in the Sciences, Leipzig, Germany; Cajal Institute, Consejo Superior de Investigaciones Científicas, Spain

## Abstract

We propose a novel, information-theoretic, characterisation of cascades within the spatiotemporal dynamics of swarms, explicitly measuring the extent of collective communications. This is complemented by dynamic tracing of collective memory, as another element of distributed computation, which represents capacity for swarm coherence. The approach deals with both global and local information dynamics, ultimately discovering diverse ways in which an individual’s spatial position is related to its information processing role. It also allows us to contrast cascades that propagate conflicting information with waves of coordinated motion. Most importantly, our simulation experiments provide the first direct information-theoretic evidence (verified in a simulation setting) for the long-held conjecture that the information cascades occur in waves rippling through the swarm. Our experiments also exemplify how features of swarm dynamics, such as cascades’ wavefronts, can be filtered and predicted. We observed that maximal information transfer tends to follow the stage with maximal collective memory, and principles like this may be generalised in wider biological and social contexts.

## Introduction

Animal groups in nature often exhibit striking examples of spatial aggregation, e.g. schools of fish, swarms of locusts, herds of wildebeest, and flocks of birds [1]–[3]. Such aggregations may provide individuals with protection, mate choices, foraging, habitat assessment, migratory routes, etc. [4], [5]. Complex large-scale patterns and structures emerge within swarms through individual decisions based on perception of local conditions. It has been observed that small perturbations cascade through an entire swarm in a wave-like manner [6], with these cascades conjectured to embody information transfer [7]. Even a few individuals may strongly bias the motion of an entire group. For instance, if a certain number of fish in close proximity turn together, this may result in a wave of turning across the whole group [8]. Formation of waves is a widespread phenomenon observed in animal groups [6], [7], [9], seeming to rapidly transfer information over long ranges. Such waves are typically conjectured as information cascades [7], and we aim to quantify these cascades in precise information-theoretic terms.

In a seminal work, Bikhchandani et al. [10] defined an “informational cascade” as a phenomenon occurring “when it is optimal for an individual, having observed the actions of those ahead of him, to follow the behaviour of the preceding individual without regard to his own information”, i.e. via an independence of an individual’s action from their private information signal. They identified two social regularities that can be explained by informational cascades: localised conformity of behaviour and fragility of mass behaviours. Their approach was not information-theoretic and did not quantify a precise information content stored/acquired within a group or transferred by a cascade.

Information cascades in collective systems often result in a rapid autocatalytic adaptive response to changing conditions [7]. This heightened response allows the group to be extremely sensitive to weak or ambiguous external stimuli, though retaining some susceptibility to noise, incorrect decisions and false alarms [5], [7], [11].

Dall et al. [12] mentioned that public information favours group cohesion, argued that information implies utility as well as uncertainty reduction, and proposed an explicit statistical decision theory framework. Their approach did not quantify either the degree of swarm cohesiveness due to public information, or information cascades *per se*. They pointed out that Shannon-Weaver entropy and similar ideas focused on simple reductions of uncertainty do not suffice in organismal biology. We argue that the *information dynamics* model used in our study goes beyond these simple ideas by utilising a directed measure (transfer entropy [13]) for information cascades, as well as localising average information-theoretic quantities.

As pointed out by Katz *et al.* [14] important questions are how animals integrate information from widely disparate sources in real time [15] and how this nonlinear integration translates into higher-order collective computational capabilities. There is an emerging understanding that information is a crucial currency for animals from both a behavioural and evolutionary perspective [12], [16]. In this work, we take an information-theoretic viewpoint on distributed computation occurring within swarms, utilising a recently introduced framework for local information dynamics.

Coherence in the swarm is ultimately related to collective *memory* (e.g. long range interaction) [15], [17], which benefits individuals locally and the “localised conformity of behaviour” becomes efficient [10]. Thus our first hypothesis is that the collective memory within the swarm that is used for computation is captured by *Active Information Storage* (AIS) [18], [19]. The local AIS of an agent in the system is the amount of information in its past that is used in predicting its next state. The overall swarm’s AIS is the average over all individuals at any given time.

Information cascades, on the other hand, are manifestations of long range *communications* that either dynamically reorganise the swarm reducing the “fragility of mass behaviour” [10] or propagate incorrect decisions [11]. Our second hypothesis is that information cascades are captured by conditional *Transfer Entropy* (TE) [20], [21], which characterises the communication aspect of distributed computation. The local information transfer between a source and a destination agent is defined as the information provided by the source about the destination’s next state that was not contained in the past of the destination [13]. Importantly, TE properly measures a directed, dynamic transfer of information.

At this stage we would like to contrast the measures of *transfer entropy* and *information flow*. These measures must be used separately to quantify information transfer and causal information flow respectively.

Transfer entropy was introduced by Schreiber [13] and has seen been applied in different settings. For instance, in computational neuroscience, the study [22] presented a novel method for interregional connectivity analysis, using multivariate extensions to the mutual information and transfer entropy. The method identified the underlying directed information structure between brain regions, highlighting changes in the structure according to behavioral conditions. The study also pointed out differences between transfer entropy and Granger causality. The main advantage is the capture of nonlinear relationships because nonlinear coupling cannot be detected by linear methods (e.g. Granger causality, nor with the non-directional mutual information).

Other relevant neuroscientific studies include the work of Wibral *et al.* [23] which utilized transfer entropy analysis of magnetoencephalography (MEG) source-level signals in detecting changes in cortical and subcortical networks between the different auditory task types, the work of Chicharro and Ledberg [24] which considers brain as a biological system consisting of multiple interacting subsystems and shows that the influence of causal connections on the natural dynamics of the system often cannot be analysed in terms of the causal effect of one subsystem on another.

*Information flow* was proposed as a measure for causal information flow by Ay and Polani [25], and it is important to realise a crucial difference between (1) transfer entropy and (2) information flow. As argued by Lizier and Prokopenko [26], predictive transfer (measured with transfer entropy) refers to the amount of information that a source variable adds to the next state of a destination variable; i.e. “If I know the state of the source, how much does that help to predict the state of the destination?”. On the other hand, causal effect (measured with information flow) refers to the extent to which the source variable has a direct influence on the next state of a destination variable, i.e. “If I change the state of the source, to what extent does that alter the state of the destination?”.

The difference between transfer entropy as a method to capture information transfer, and information flow as a measure to capture causal effect/flow, is very important and may cast observations in a different light. In this work, we stay completely within the interpretation of predictive information transfer, and do not make any claims on detecting causal information flows.

Memory typically refers to the storage of information by an agent or process to be used in its future. It can be understood in a wider (collective/distributed) context, where stigmergy is used as a means to share information between agents via environment [18]. Grassé [27] introduced the term stigmergy (“previous work directs and triggers new building actions”) to describe a decentralised pathway of information flow in social insects. Stigmergy is a mechanism of indirect coordination among agents acting in the environment, where local traces left in the environment by decentralised actions stimulate the performance of subsequent actions by the same or a different agent. In a more applied sense, Klyubin *et al.* [28] treated agent’s sensors as extracting information and actuators as having the capability to “imprint” information on the environment, thus viewing agents as creating, maintaining and making use of various information “flows”. For example, the individuals within a swarm can put some information out into the environment, then retrieve it at a later point in time by sensing –– i.e., individuals do not have to keep all of the information internally and can share a distributed collective memory through interactions with the environment or other individuals.

One may take a causation approach to measuring memory by computing causal information flows using interventionist approach of Ay and Polani [25]. In other words, one would attempt to impose on source variables and determine the changes in the destinations brought about by these impositions. For instance, if a swarm model is described by differential equations, one may estimate the effects of interactions between individuals by modifying terms of the model. In this work, however, we take a simpler approach to measuring memory via information storage, without causal flows.

To re-iterate, we hypothesise that AIS captures the active/predictive collective memory within the swarm while TE measures information cascades. To verify these hypotheses, we explore two scenarios. Our first experiment checks how different local initial perturbations affect a single swarm. The second experiment introduces a different type of perturbations, brought about by three separate but merging swarms. We use a Lagrangian model for modelling and simulating aggregations of discrete individuals. Each individual responds to its neighbours in three concentric zones with repulsion, orientation, or attraction, respectively [29]–[32]. The experiments quantitatively confirm our conjectures by tracing AIS and TE over time. The observed local and global maxima of these measures allow us to identify different elements of swarm dynamics (see Movie S1, S2, S3, S4 for the videos).

## Results

**Figure 1:**
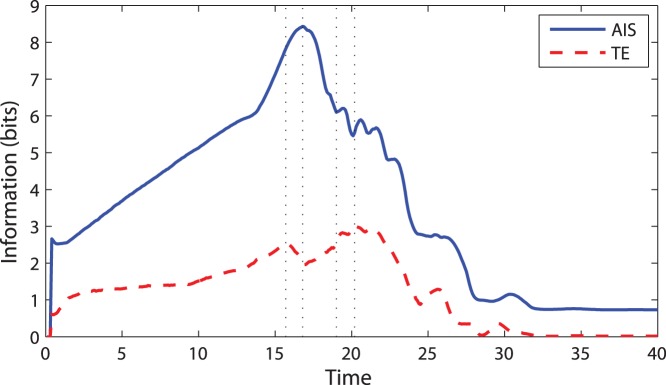
Information storage and transfer over time for a swarm initially in a square configuration. Shown here are the average active information storage (AIS) per particle and average transfer entropy (TE) per particle pair.

Initially, the individuals in the centre of the swarm are not affected by changes at the swarm’s periphery. As the changes propagate deeper, more and more individuals get engaged in collective computation and the collective memory grows, creating coordinated motion. When the majority of individuals are dynamically coordinated, average AIS of the swarm reaches its maximum (Fig. 1 at 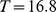).

**Figure 2:**
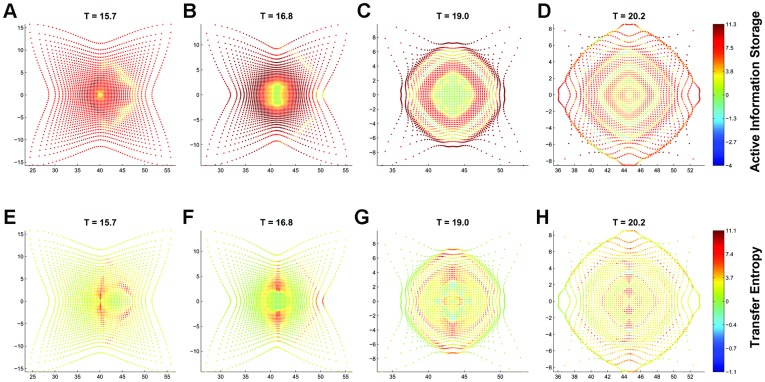
Local information dynamics for a swarm initially in a square configuration. (Top) Local average information storage through the swarm at key time steps. (Bottom) Local average information transfer in a swarm at key time steps. The individuals’ two-dimensional positions are plotted on the 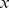 and 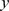 axes, and their colours represent the AIS and TE values in bits, as shown by the scale on the colour bar. Note that the axes scales are adjusted, and the colours are scaled differently for positive and negative values.

Figures 1, 2 and Movie S1 trace information dynamics over time, and show that local AIS can be positive and negative. Positive local AIS indicates that the past informs about the next state, while negative values indicate that the past misinforms about the next state [18], [21]. Negative local storage means that an individual’s movement is unusually strongly influenced by other individuals (via high transfer) at this time, given the past history of that individual.

We argue that negative local AIS represents processing of new data propagated from elsewhere. For example, in Fig. 2C some of individuals in the centre are trying to compute their next state while being influenced by competing signals from their neighbours. In such situations, their past is misinformative about the next state. This results in the overall AIS decrease from the maximum. This decrease may be interrupted when the misinformed individuals are outnumbered by the individuals moving coherently. However, as the swarm synchronies to a steady motion, AIS (i.e. active collective memory) converges to a positive constant near zero. This can be seen by writing the joint probability 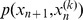 as 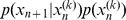 according to Bayes’ Rule, making the log term in Equation 6 equal to 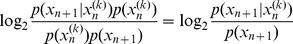. When the swarm is in steady motion, 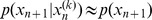, making the log term approximately zero. The ‘bell’ shaped curve of AIS is reminiscent of many complexity curves [33], [34], indicating that the most complex collective behaviour can be characterised information-theoretically.

Now we turn our attention to the communication aspect of computation, modelling information cascades by TE. As the swarm begins to ‘absorb’ the initial changes originated at the periphery, the first wave results in slightly increasing overall TE (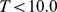). The moment the wave reaches the centre, some individuals there acquire high local TE, being strongly influenced by their neighbours. Their new dynamics generates a new information wave spreading outwards through the swarm, achieving a local maximum at 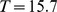 followed by a local minimum when it dissipates at 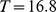. At this time AIS attained its global maximum, and the computation is non-trivial involving both memory and communication.

Local TE can also be positive or negative [20], [21] (Fig. 2, bottom row, and Movie S2). Positive local TE means that the source agent is informative about the next state of the destination, given the destination’s history (the movement is strongly affected by its neighbours). Negative TE indicates that the source misleads an observer (when the individual is either exhibiting strong independent motion or is under the collective influence of several neighbours rather than the coherent influence of a single neighbour, e.g. 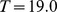). These information dynamics suggest that transfer alternates with storage. Indeed, Figure 2 shows in most cases, areas of high local storage often have low or negative local transfer and vice versa.

Individuals that begin to move coherently (i.e. have comparably high local TE) form a front of a cascade as seen at 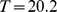. At that time, TE reaches its global maximum because the formed cascades dominate incoherent individuals. Not surprisingly, this stage has followed the time when memory (AIS) was highest. Eventually, the cascades help to coordinate the swarm, creating a steady configuration. TE decreases as the swarm ‘crystallises’.

**Figure 3:**
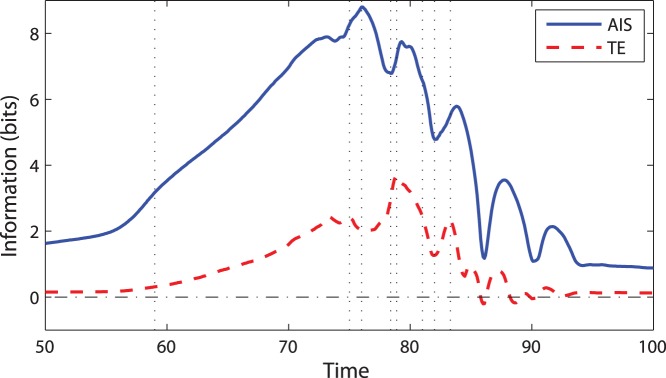
Information storage and transfer over time for a swarm initially consists of three squares in a checker configuration. Shown here are the average active information storage (AIS) per particle and average transfer entropy (TE) per particle pair.

**Figure 4:**
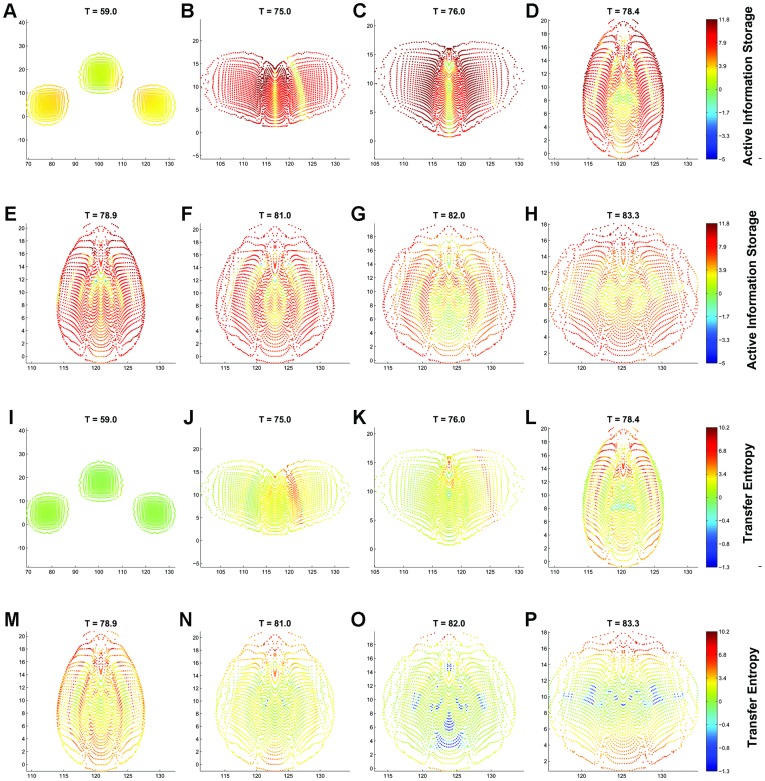
Local average information dynamics in a swarm at key time steps. A-H, active information storage, I-P, transfer entropy. The swarm initially consists of three squares in a checker configuration. The individuals’ two-dimensional positions are plotted on the 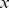 and 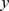 axes, and their colours represent the AIS and TE values in bits, as shown by the scale on the colour bar. Note that the axes scales are adjusted, and the colours are scaled differently for positive and negative values.

Our second experiment (Fig. 3 & 4, and Movie S3 & Movie S4) models three swarms that eventually start interacting with each other. Thus, this experiment allows us to model different boundary perturbations.

When the swarms start to interact and the individuals become more dynamically coordinated, the overall local storage increases until it reaches global maximum at 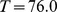 (Figure 4A–C). This is the moment when all three swarms merge into a single coordinated entity, confined within a spatial extent that will not change significantly past this point. Importantly, the majority of individuals are dynamically coordinated at this instant, which is followed by several ‘waves’ (Figure 4D–H) that interleave groups of individuals with higher and lower local AIS. This process non-monotonically reduces AIS, while the swarm converges to the state of synchrony, with AIS being near zero (similar to the first experiment).

Similar to the first experiment, as the swarms merge (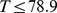), the overall TE is mostly increasing because there are several significant information cascades ‘rippling’ through the swarm. This shows that as the swarm is merging into one group, the specific individuals are under the most influence from their neighbours. The maximum TE lags behind that of AIS (analogously to the first experiment) again highlighting rich computation in terms of both collective communication and memory.

Tracing TE reveals some new features. Firstly, when the swarms merge, while moving from left to right, we can observe asymmetry in local values: a front of negative local TE on the left (where individuals were ‘surprised’ because the direction of dominant attraction was roughly opposite to the current direction of the overall swarm motion), and a front of positive local TE on the right (where these two directions concurred).

The second new feature is propagation of an initial asymmetry in swarms. For example, negative local TE at 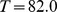 (Fig. 4O) are particularly visible. As the swarm progresses towards a steady state, there are further local fluctuations reducing the overall information storage and transfer values, showing that the distributed computation declines.

## Discussion

The reported results provide the first quantitative evidence (verified in a simulation setting) with a direct measure of information for the long-held conjecture that the information cascades occur in waves rippling through the swarm. The cascades can be observed via coherent changes in local TE, and are akin to information cascades in other systems, e.g. gliders in cellular automata [20]. Our characterisation deals with weak and ambiguous external stimuli by incorporating both positive and negative local TE. In contrast to previous studies, information cascades are not just observed as changes in behaviours and activities, but are rather rigorously determined and computed.

In addition, we introduced a novel information-theoretic characterisation of swarm’s collective memory, which is identified with AIS. Higher values of AIS are associated with higher levels of dynamic coordination. This study reveals different ways in which a particle’s spatial position is dynamically related to its information processing role.

Collective communication and memory are two necessary elements of distributed computation (in addition to information modification [21], [35]). The information-theoretic approach clearly separates different elements of distributed computation taking place in swarms, filtering and predicting important hot spots (e.g. a cascade’s wavefront, collective memory’s core, etc.). In addition, this framework may reveal new biological/social principles that govern coherent aggregation of living organisms (e.g. maximal information transfer tends to follow the stage with maximal collective memory).

## Methods

We use a three-zone swarming model that features continuous, concentric circular and overlapping zones with smooth transitions. In an appropriate limit corresponding to a swarm consisting of a large number of individuals, the dynamics of the system is governed by a system of partial differential equations describing the density and velocity of the swarm [36]. To perform simulations, the density and velocity fields are systematically discretized into individuals with two-dimensional position vector 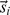, velocity 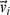 and acceleration 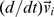. For this model, individuals turn toward a desired direction,
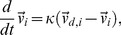
(1)where 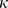 is a turning rate parameter and 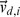 is the desired direction of the 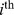 individual. The desired direction is a linear combination of influences,
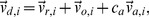
(2)where 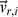, 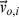 and 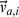 are the influences from the zones of repulsion, orientation and attraction, respectively and are given by:



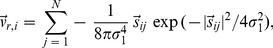
(3)

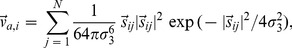
(4)and
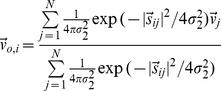
(5)where 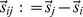, the relative position of individuals 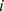 and 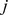. The lengths 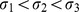 represent the sizes of the repulsion, orientation and attraction zones, respectively. The parameter 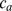 in Equation 2 controls the relative importance of attraction over repulsion. An individual will be influenced to move away from other individuals that are within the innermost zone of repulsion, to align with individuals in the central zone of orientation and to move toward individuals in the outer zone of attraction. The constant 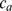 specifies the importance of attraction relative to orientation and repulsion.

We integrated the individual trajectories using the scipy.integrate.odeint Python package so that they are numerically resolved to a relative error of 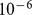. To construct time series, we subsampled the trajectories at time intervals of 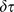. We start our investigation with individuals in a square configuration 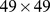 in size, or individuals in three squares of checker configuration initially, each square is 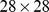 in size.

AIS for agent 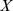 is the local mutual information from its semi-infinite past 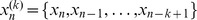 (as 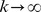) to its next state 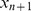 at time step 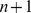 [18]:
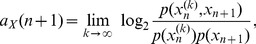
(6)with 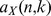 representing an approximation with finite history length 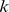. The overall AIS is the average 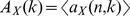.

The local TE [20] from a source agent 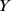 to a destination agent 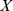 is the local mutual information between the previous state of the source 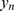 and the next state of the destination 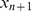, *conditioned* on the past of the destination 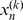. In this study, we also condition it on another contributor 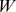 to form the *conditional transfer entropy* [21]:
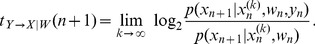
(7)Again, 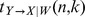 represents finite-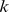 approximation, and the overall TE is the average: 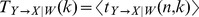.

To apply information dynamics to swarms, we accumulated the observations across agents and measured the state transitions with relative variables [37]. For local AIS, the variables in Eq. 6 are: 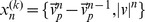, and 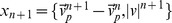. For TE, we do not take into account the speed in 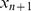, and 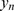 is the relative positions and velocities between two individuals, thus, 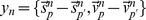, 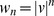, 
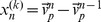
, and 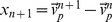.

For each individual, we compute local TE from all neighbours within a certain radius and average these values into the local TE for that individual. While each TE could be viewed as akin to a vector, i.e. having magnitude and relative direction from the source to the destination, these components are quite separate and the total information transferred to an individual does not add together in a simple vector-wise fashion. (Indeed, it adds together properly by considering incrementally conditioned transfer entropy terms, see [21]). As such, it is more meaningful to consider the average information received by the individual from each source. The average TE for the swarm is the average of these averages. For example, Figs. 2E–H show the local TE values for individuals at different times, while Fig. 1 traces the swarm average over time.

## Supporting Information

Movie S1
**Local active information storage (AIS) of swarm individuals over time for experiment 1.** We start the simulation with the swarm individuals in a square configuration 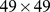 in size. The top panel shows the average AIS per individual as depicted in Figure 2 in the main text, with the vertical line highlighting the specified time. The bottom panel shows the swarm dynamics at the specified time, the individuals’ two-dimensional positions are plotted on the 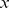 and 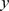 axes. The local AIS values for each individual is shown here in different colours, according to the scale on the right. Note the colours are scaled differently for positive and negative values.(MOV)

Movie S2
**Local transfer entropy (TE) of swarm individuals over time for experiment 1.** We start the simulation with the swarm individuals in a square configuration 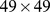 in size. The top panel shows the average TE per particle pair as depicted in Figure 0 in the main text, with the vertical line highlighting the specified time. The bottom panel shows the swarm dynamics at the specified time, the individuals’ two-dimensional positions are plotted on the 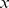 and 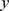 axes. The local TE values for each individual is shown here in different colours, according to the scale on the right. Note the colours are scaled differently for positive and negative values.(MOV)

Movie S3
**Local active information storage (AIS) of swarm individuals over time for experiment 2.** We start the simulation with the swarm individuals in three squares of checker configuration; each square is 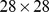 in size. The top panel shows the overall AIS as depicted in Figure 2 in the main text, with the vertical line highlighting the specified time. The bottom panel shows the swarm dynamics at the specified time, the individuals’ two-dimensional positions are plotted on the 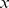 and 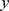 axes, with the colours of each individual denoting the value of its local AIS. The local AIS values for each individual is shown here in different colours, according to the scale on the right. Note the colours are scaled differently for positive and negative values.(MOV)

Movie S4
**Local transfer entropy (TE) of swarm individuals over time for experiment 2.** We start the simulation with the swarm individuals in three squares of checker configuration; each square is 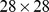 in size. The top panel shows the overall TE as depicted in Figure 4 in the main text, with the vertical line highlighting the specified time. The bottom panel shows the swarm dynamics at the specified time, the individuals’ two-dimensional positions are plotted on the 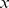 and 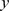 axes, with the colours of each individual denoting the value of its local AIS. The local AIS values for each individual is shown here in different colours, according to the scale on the right. Note the colours are scaled differently for positive and negative values.(MOV)
